# Supplementary material for: Seismic precursors to the Whakaari 2019 phreatic eruption are transferable to other eruptions and volcanoes
Source: Nat Commun. 2022 Apr 20;13:2002. doi: 10.1038/s41467-022-29681-y (PMC9021187; doi:10.1038/s41467-022-29681-y)
Supplement: Supplementary file 1 — Supplementary Information [file 41467_2022_29681_MOESM1_ESM.pdf]

SUPLEMENTARY INFORMATION

Supplementary Note 1

Quick recipes

The steps below describe calculation of precursors discussed in this study.

The first step is to calculate the data stream. There are several sub-steps:

(1) After removing the instrument response to the seismic signals, apply a bandpass filters to each 24 hours of data, between 2-4.5, 4-8 and 8-16 Hz (corresponding to the RSAM, MF and HF bands).
(2) Compute the absolute values of each signal.
(3) Subdivide the signals into 10 minutes intervals. For each interval, compute the average value as the RSAM, MF and HF datapoints assigned to that interval.
(4) Removing outliers associated with regional earthquakes is optional. We procced as follow: from (2), subdivide the signals into 10 minutes intervals. Calculated the mean and standard deviation (mu and sigma) for each interval. Apply z-score normalization in log-space to the interval using mu and sigma. Check if any value in the interval exceeds a threshold of 3.2 standard deviations above the mean. If yes, exclude data points from a 150s mask starting 15s before the outlier located. Calculate the average value in the interval excluding points inside the mask: this the RSAM, MF and HF value for the interval.

To calculate the DSAR, procced as follow:

(1) Integrate the bandpass filtered MF and HF data with time.
(2) Take the absolute value and compute averages on 10-minute intervals.
(3) Compute the ratio between integrated MF and HF.

For computing the DSAR median feature proposed as a precursor in this paper follow:

(1) Every 10 minutes in the DSAR data stream, take a 48 hours window (looking backwards).
(2) For each window, compute the median.
(3) Construct the feature time series with point every 10 minutes corresponding the medians computed from the 48 hours windows.

The second precursor corresponds to the DSAR rate variance (named for the tsfresh feature that measures the change quantiles variance between percentiles .4 and .6) and is calculated:

(1) Every 10 minutes in the DSAR data stream, take a 48 hours window (looking backwards).
(2) For each window, compute the corresponding percentiles 0.4 and 0.6.
(3) For data points between the percentiles (in each window), calculate the difference between subsequent pairs (obtaining a vector of differences).
(4) Compute the variance in the differences vector.

Supplementary Tables

Volcano	*Country*	*Station*	*Network*	*# rec. eruptions*	*Type or eruptions*	*Eruptions year*	*Record years*	*Continuous data*
**Pavlof**	Alaska, USA	PVV 4 km	AV	3	Magmatic	14, 14,16	2 .5	Yes 69%
**Veniaminof**	Alaska, USA	VNSS 5.3 km	AV	2	Magmatic	13,18	4	Yes 59%
**Bezymianny**	Kamchatka, Russia	BELO 1 km	YC	3	Magmatic	07,07,07	1	Yes 99%
**Whakaari**	New Zealand	WIZ 500 m	NZ	5	Phreato-magmatic	12,13,13, 16,19	11	Yes 96%
**Tongariro**	New Zealand	KRVZ	NZ	2	Phreatic	12,12	14	Yes 96%
**Ruapehu**	New Zealand	FWVZ	NZ	3	Phreatic	06,07	14	Yes 91%

Table S1. Basic information on the six volcanoes included in this study indicating the country, the station and network used and its distance to the crater, the number of eruptions recorded, the type and year of eruptions, the length of the seismic record analysed, and the % of continuous data on the record.

**Eruption**	**Time (UTC)**	**VEI**	**Type**	**Duration**	**Column Height**
**Whakaari, 2012**	2012 08 04 16 52 00	1	Phreatic	10 min	
**Whakaari, 2013a**	2013 08 19 22 23 00	0-1	Phreatic	10 min	
**Whakaari, 2013b**	2013 10 11 07 09 00	0	Phreato-magmatic	10 min	
**Whakaari, 2016**	2016 04 27 09 37 00	1	Phreatic		
**Whakaari, 2019**	2019 12 09 01 11 00	2	Phreatic	2 min	
**Ruapehu, 2006**	2006 10 04 09 30 00	0	Phreatic		
**Ruapehu, 2007**	2007 09 25 08 20 00	1-2	Phreatic		~5 km
**Ruapehu, 2009***	2009 07 13 06 30 00	0	Phreatic		
**Tongariro, 2012a**	2012 08 06 11 50 00	2	Phreatic		
**Tongariro, 2012b**	2012 11 21 00 20 00	1	Phreatic		
**Pavlof, 2014a**	2014 05 31 17 22 00	3	Magmatic	1 month	~7 km
**Pavlof, 2014b**	2014 11 13 00 00 00	2	Magmatic	5 days	~11 km
**Pavlof, 2016**	2016 03 28 01 33 00	3	Magmatic	7 days	~10 km
**Bezymiany, 2007a**	2007 09 25 08 30 00	3	Magmatic		~4 km
**Bezymiany, 2007b**	2007 10 14 14 27 00	2	Magmatic	1 day	~9 km
**Bezymiany, 2007c**	2007 11 05 08 43 00	3	Magmatic		~8 km
**Veniaminof, 2013**	2013 06 13 00 00 00	2-3	Magmatic	4 months	~6 km
**Veniaminof, 2018**	2018 09 04 00 00 00	2	Magmatic	4 months	~5 km

Table S2. Catalogue of eruptions included in this study, indicating its time, volcano explosivity index (VEI), eruption type and duration, and column height. See Figure S1. Ruapehu 2009 July 13 wasn't reported as an eruption but as phreatic activity by Geonet (Scott 2013; https://www.gns.cri.nz/static/pubs/2013/SR%202013-045.pdf). By inspection of the available data (seismic, lake temperature, lake level, and rainfall data; see Figure S10), we consider that it might be a missed small eruption given the sharp increase in the lake level (without rain) and a significant peak in the RSAM (MF and HF data too) (given the presence of the lake that could obscure small events, missing small eruptions in Ruapehu is reasonable). By closer inspection, we found that a series of high amplitudes nDSAR median cycles occurred before the event (Figure S2). Based on these attributes, we decided to incorporate it into the analysis. However, we acknowledge that this is not recognised as an eruption event by GeoNet.

Phase	(1) Magma-hydrothermal system interaction	(2) Pulsating gas flux	(3) Sealing consolidation	(4) Pressurization	(5) Seal breakdown and Eruption
**Period**	Nov 11 - Nov 22<br>~10 days	Nov 22 - Dec 02<br>~10 days	Dec 02 - Dec 06<br>~4 days	Dec 06 - Dec 09<br>~4 days	Dec 09 01 11 00<br>~16 hours
**Observations from seismic data**	Harmonic tremor in RSAM band (2-5 Hz). Intense for 10 days. Weak for 1 month (Figure S7).	Relative increase in MF (4-8Hz) and HF bands (8-16Hz). Relative decrease in RSAM band. Cyclic pulses in the data. Increase in DSAR median. Peak in DSAR rate variance at the end of the cycle.	No pulses in seismic data. Decoupling of MF and HF. MF increases quicker than HF, causing an increase in DSAR median.	Decrease in DSAR median, due to a rapid decrease in MF. Peak in every frequency during decrease of DSAR median (~16 hours before eruption). Inverse RSAM shows a peak and a linear decrease (~16 hours before eruption).	Strong peak in every frequency of the data at the eruption time. Then the signals decrease for 1 day, followed by an increase activity for 4 days (between Dec 10 and Dec 13/14). The system remains with lower activity after that (Figure S10).
**Volcanic Alert Bulletin (VAB; Geonet)**	Change in geothermal system; surface geysering; lake level increased. VAL is raised to 2. The monitored parameters continue to be in the expected range for moderate volcanic unrest and associated hazards.	More frequent gas emissions and jetting. No lake level change. The monitored parameters continue to be in the expected range for moderate volcanic unrest and associated hazards exist.	-	-	An eruption occurred at ~14:11 local time as an impulsive, short-lived event and affected the crater floor. Activity appears to have diminished since the eruption. Our monitoring data shows that there was a short-lived eruption that generated an ash plume to ~12,000ft above the vent.
**External corroboration**	Volcanic tremor due to interaction between magma conduit and groundwater system in stratovolcanoes (days to weeks) is a rich source of pressure perturbations: system then enter to pulsation ('alive') (Chouet, 1996; Chardot et al. 2015).	Increase in SO_2 flux as pulsatory gas fluxes. Strong increase on Dec 02 (Burton et al., 2021). During eruptive periods strong correlations are observed between degassing and tremor, with a significant contribution of frequency signal in tremor around ~7 Hz (Salerno et al., 2018).	No detection of SO_2 peaks during this period. Seal formation is proposed, not specifically during this period (Burton et al., 2021).	Continuous fumarole activity (degassing), system partially sealed (Burton et al., 2021). Strong increase in gas flux (~1hour before eruption) that could have broken the seal (Burton et al., 2021).	Precursors have been observed in 2019, and both 2013 Whakaari eruptions, as strong RSAM bursts—paroxysms— some 4 h in duration, and overlaid by a shorter, 40-min oscillation. The 4-h paroxysmal signal could indicate rising magma into the lower conduit, which further charges the hydrothermal fluids. (Dempsey et al., 2020)
**Hypothesis**	Interaction between magma and the hydrothermal system causes the harmonic tremor.	Episodic pulses of magmatic degassing that cause sharp fluctuations in the in data (MF more sensible and HF due to several surface activity).	Seal consolidation (low permeability increase; less fluids or gases moving up).	System is sealed and accumulates pressure beneath it. This causes a decrease in HF (less surface activity) and in MF (less gas rising to the surface). System becomes 'quiet'.	Seal breaks due to a perturbation on a critically pressurized system. Eruption is trigger by a gas flux pulse that cause a cascading material failure that leads to an explosive eruption.

Table S4. Description of the proposed phases leading to the Whakaari 2019 eruption.

Supplementary Figures

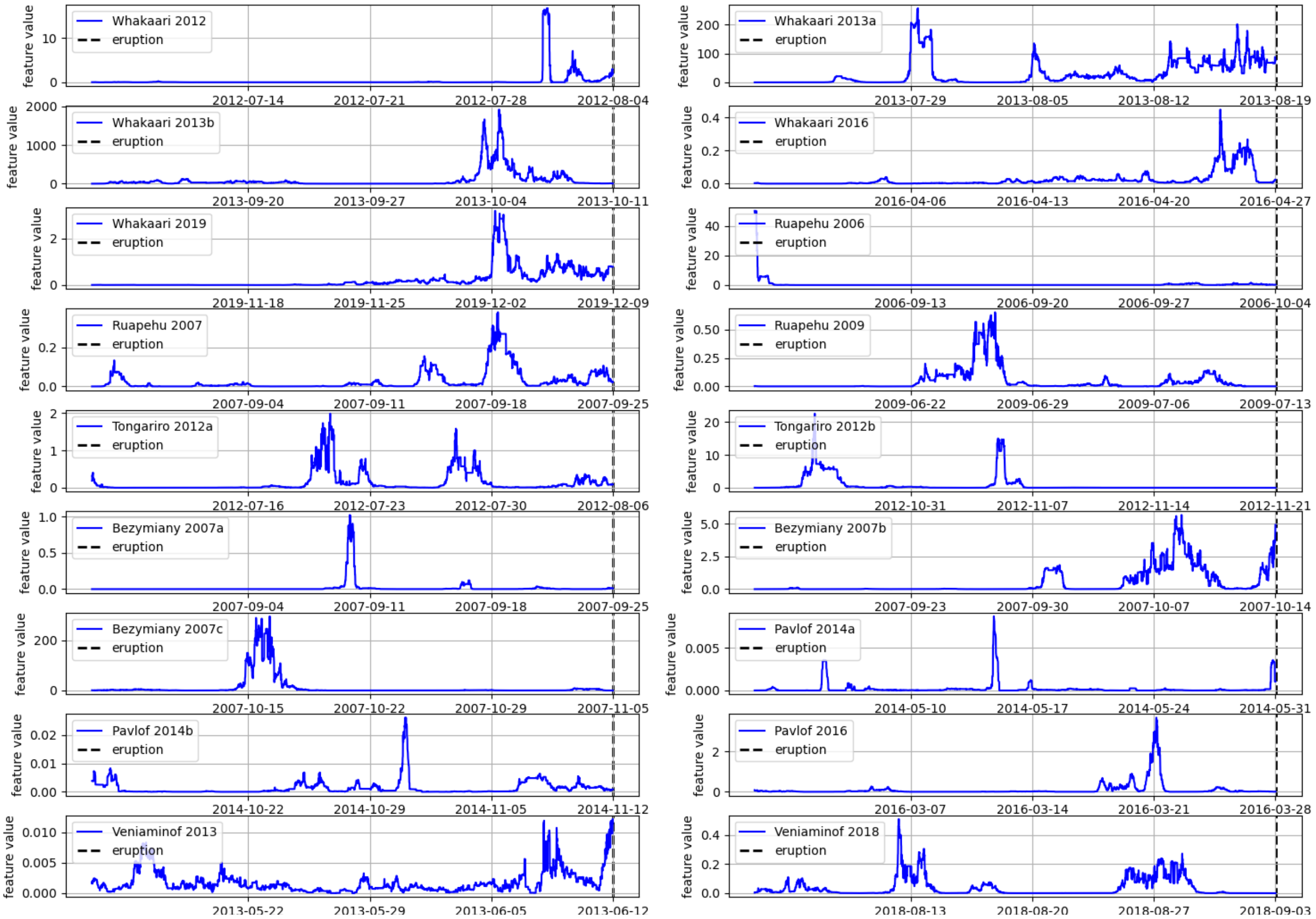

Figure S1. Subplots show the pre-eruptive one-month feature time-series for the 18 eruptions for the feature *DSAR rate variance* (*DSAR change quantiles variance (0.6-0.4)).*

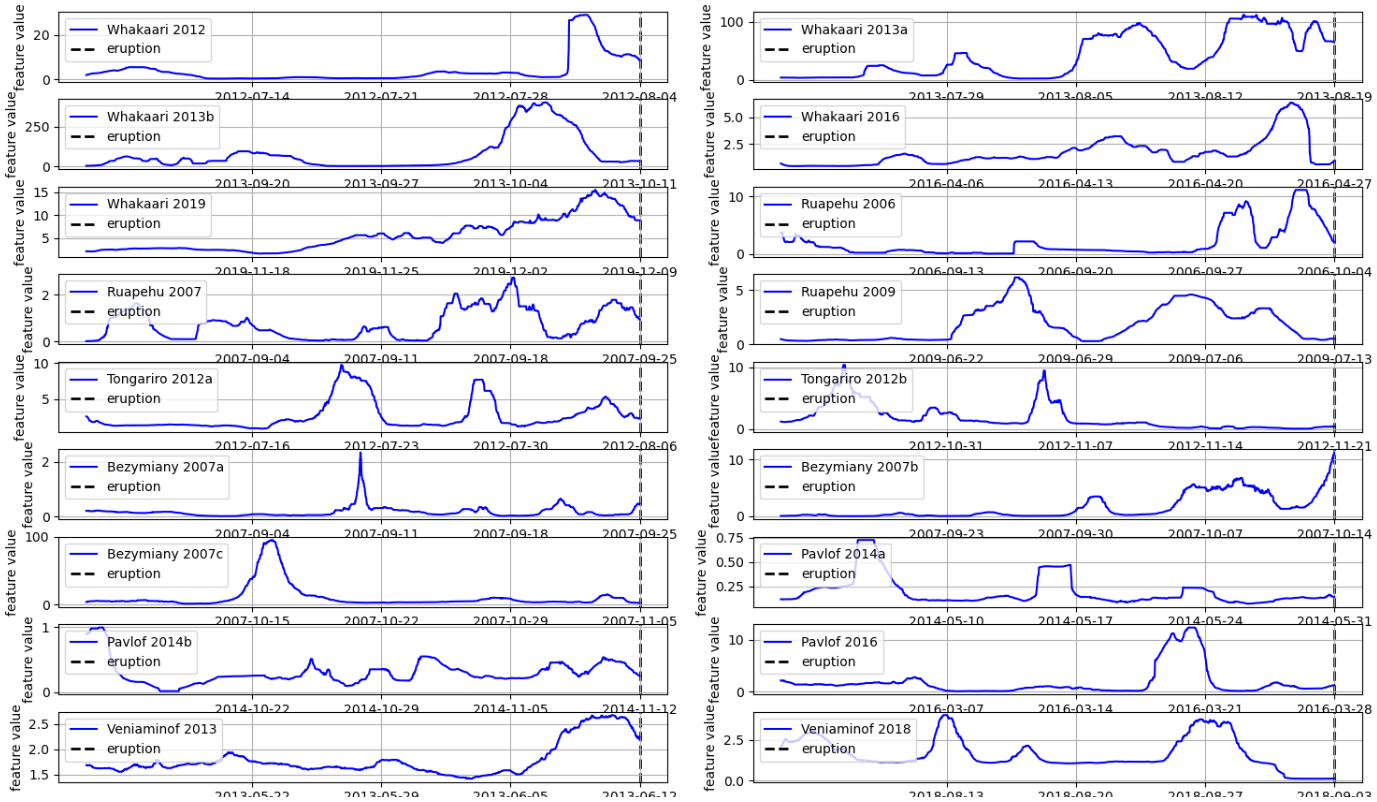

Figure S2. Subplots show the pre-eruptive one-month feature time-series for the 18 eruptions for the feature *DSAR median.*

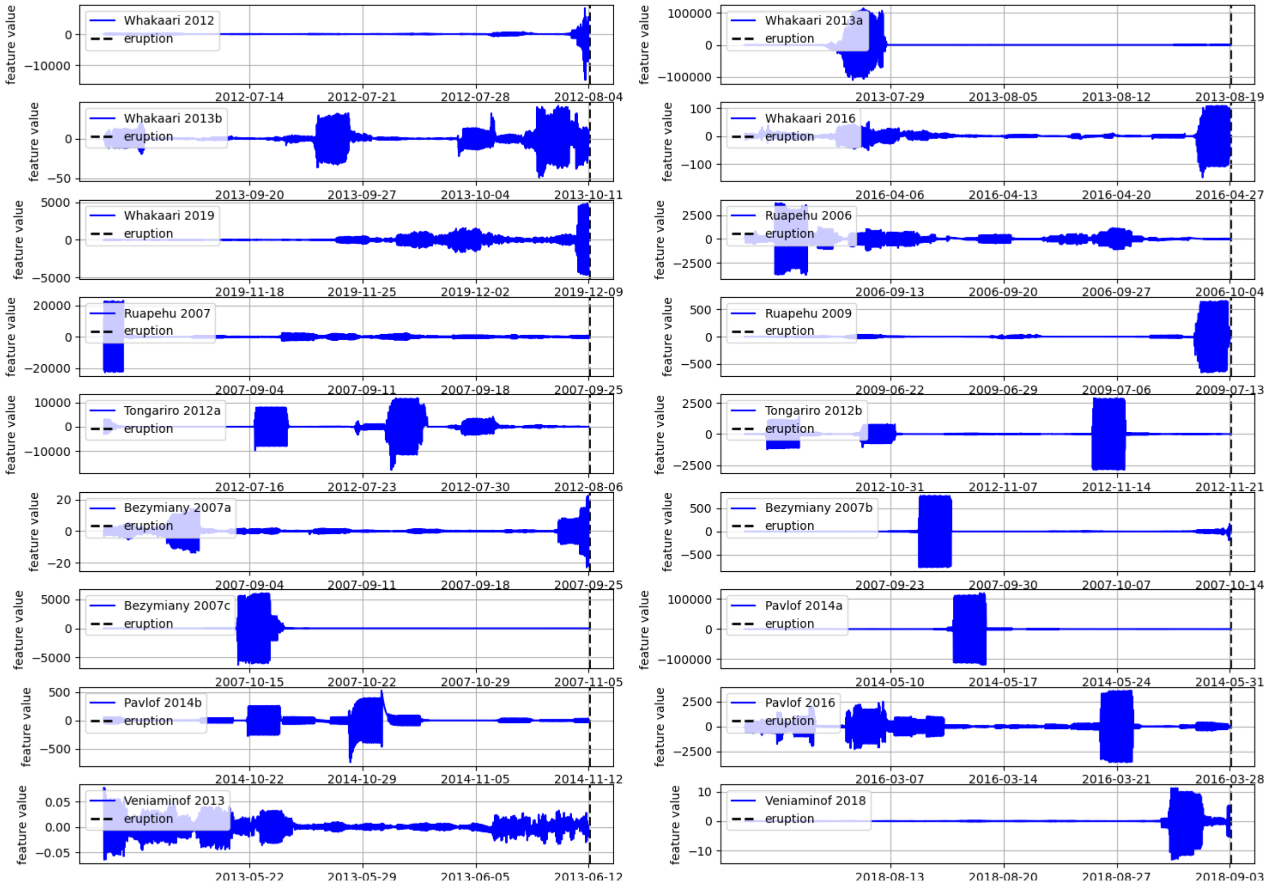

Figure S3. Subplots show the pre-eruptive one-month feature time-series for the 18 eruptions for the feature *75-minute HF harmonic (HF Fourier coeff 38).*

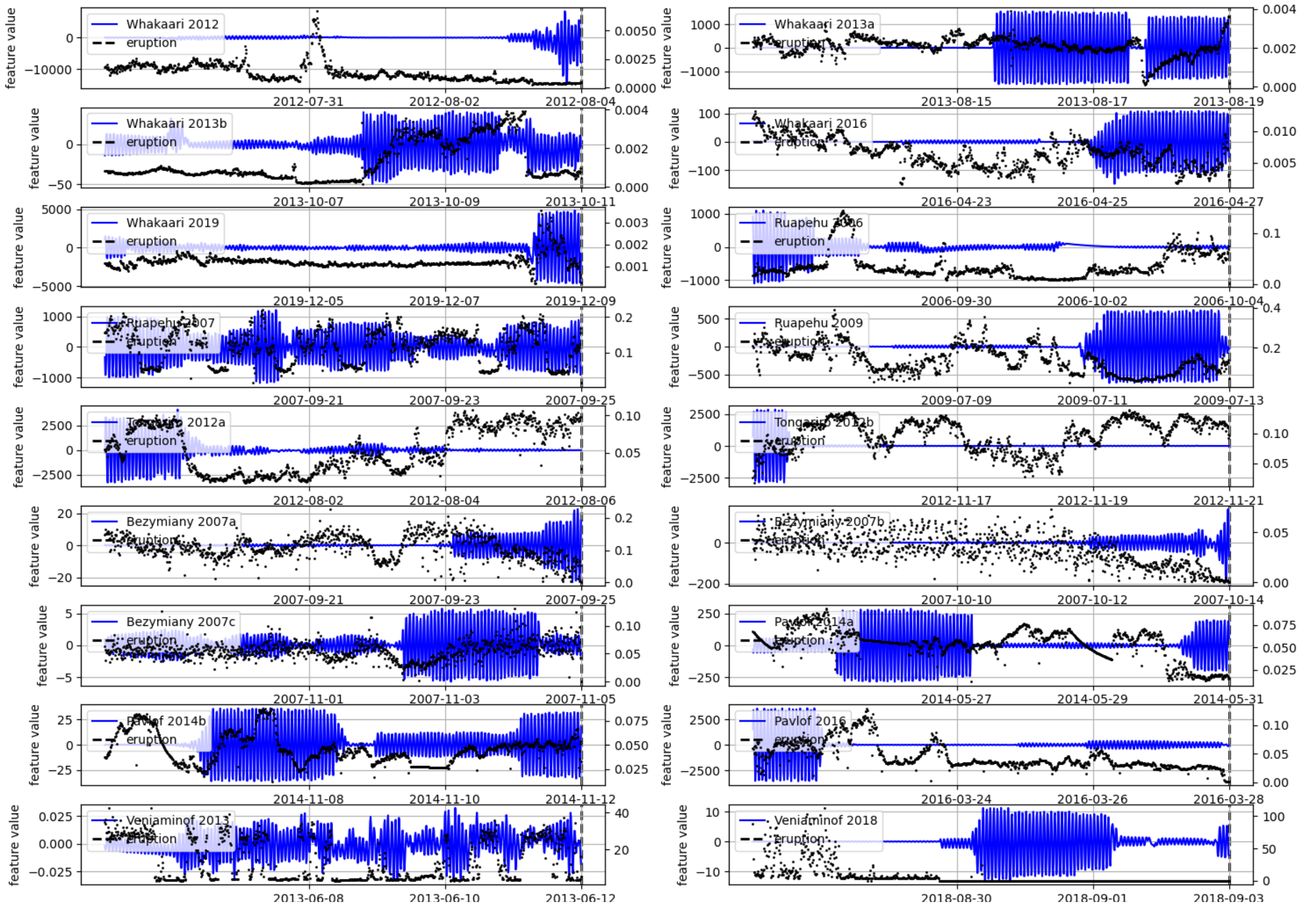

Figure S4. Subplots show the pre-eruptive two-weeks feature time-series for the 18 eruptions for the feature *75-minute HF harmonic (HF Fourier coeff 38),* along with the inversion of the RSAM data (black dots).

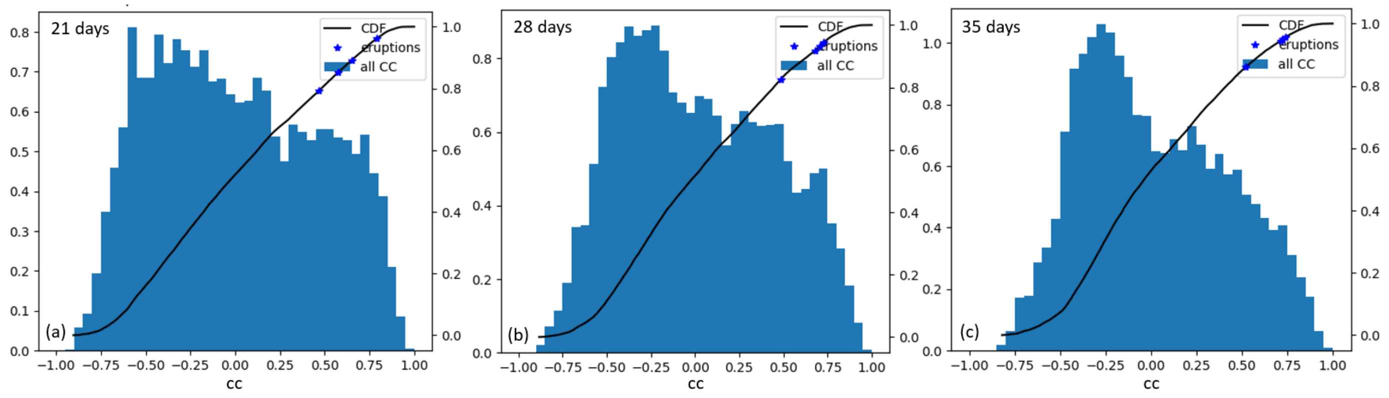

Figure S5. Statistical test similar to Figure 3a-c (see Results, Differentiability of eruption precursors) using as archetype feature time series prior to the 2019 Whakaari eruption for 3 different prior time: (a) 3 weeks, (b) 4 weeks, and (c) 5 weeks. The tests are performed over the Whakaari ~10-year record. Figures shown are equivalent to Figure 3a (30 days), but histograms are generated using convolutions (instead of shifting forward one day and correlate; see Methods).

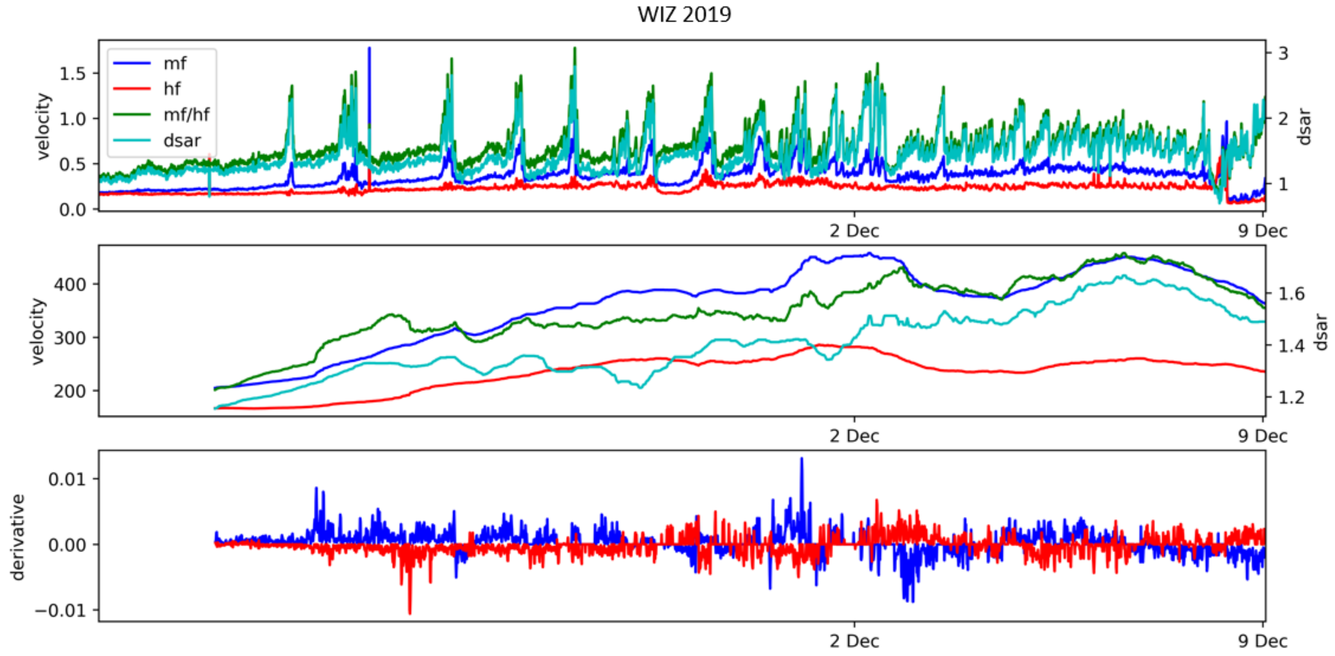

Figure S6. HF, MF and RSAM data streams one month prior to the Whakaari 2019 eruption and their rates of change.

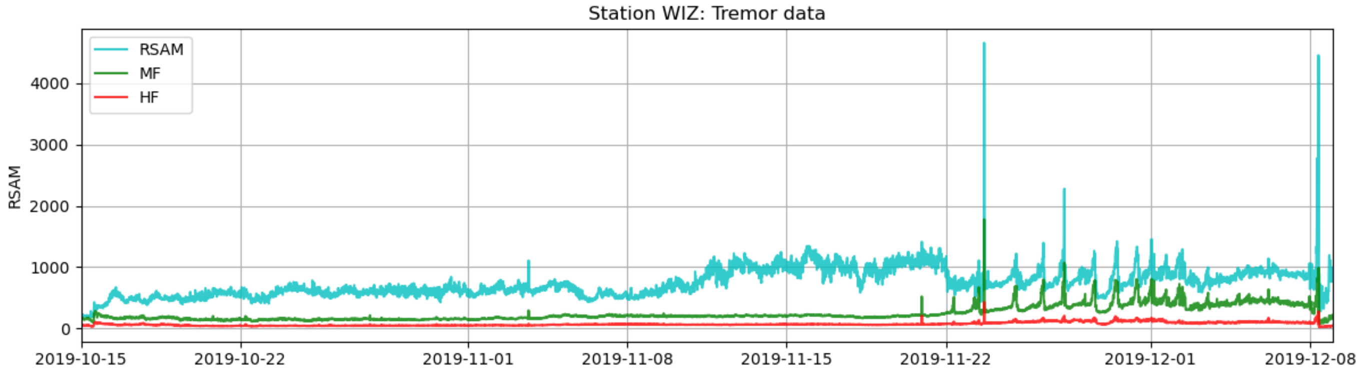

Figure S7. HF, MF and RSAM data streams 53 days before the Whakaari 2019 eruption (from the beginning of the harmonic tremor observed in the RSAM data).

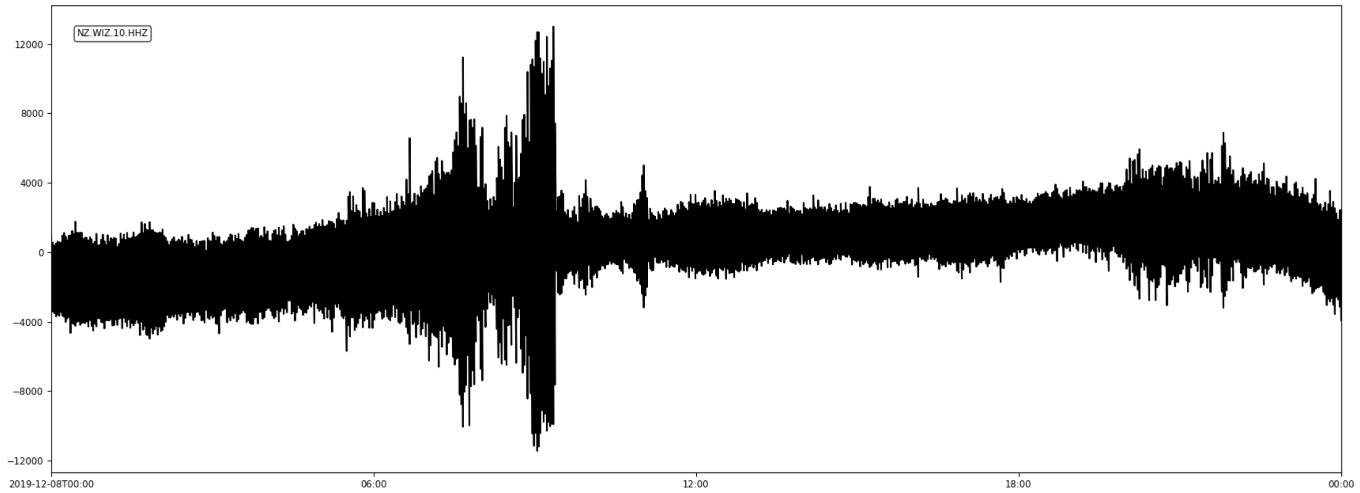

Figure S8. Raw seismic waveform of the peak observed in all data streams 16 hours before the Whakaari 2019 eruption.

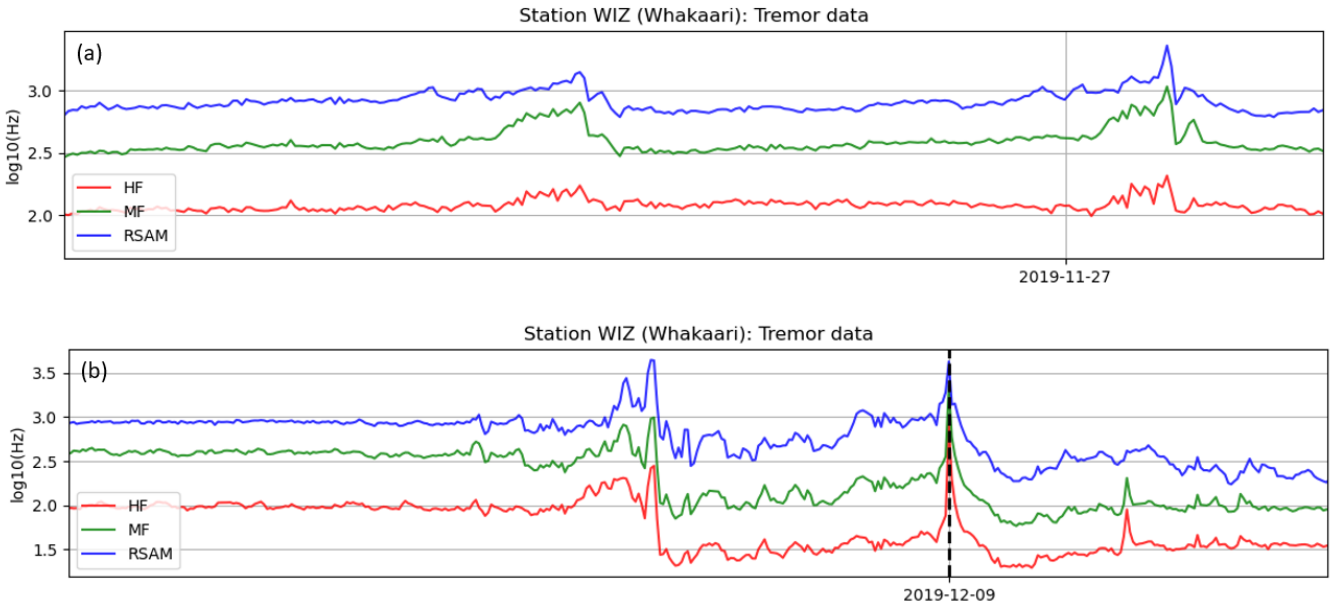

Figure S9. Data streams around the gas flux pulses during phase 2 (a) and phase 4 (b) that preceded the Whakaari 2019 eruption (dash black line).

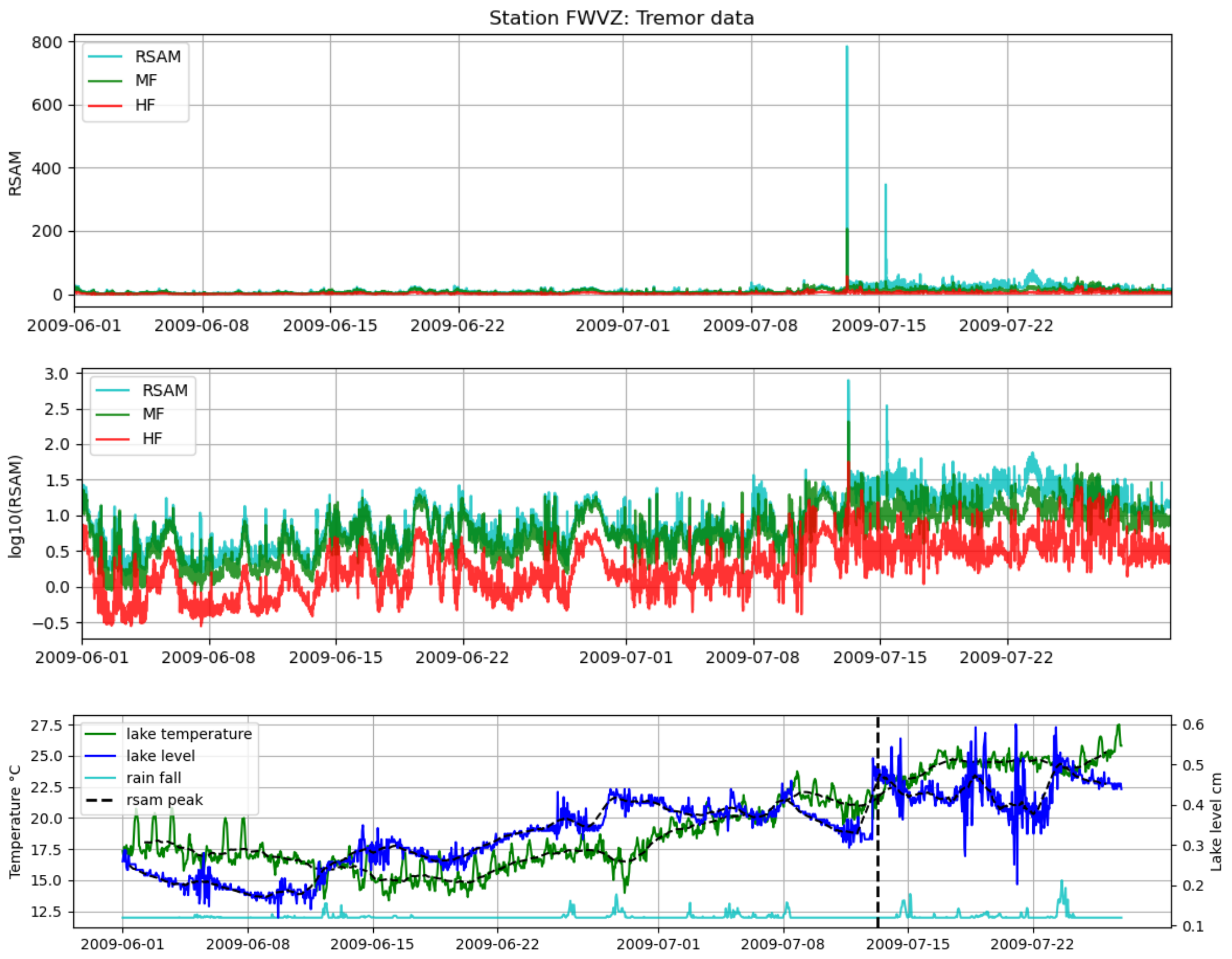

Figure S10. Datastreams (RSAM, MF, HF, DSAR) for station FWVZ in Ruapehu between the 1th and the 25th of June of 2009, where a high peak in the data suggesting a small eruptive event is highlighted. Bottom panel shows the crater lake level and temperature data for the same period, along with the rainfall data for the region.

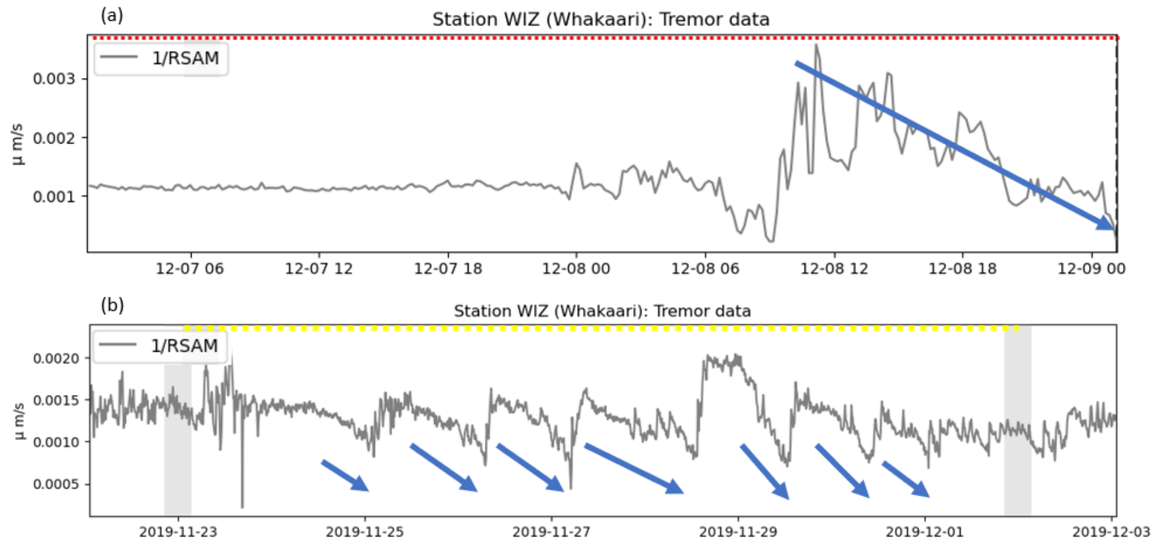

Figure S11. Inverse RSAM for station WIZ in Whakaari (2) two days before the eruption on the 9th of December 2019 (during pressurization and seal breakdown phases, indicated by the red and black dash lines) and (b) between the 23rd of November and the 3rd of December of 2019 (during pulsating gas flux phase, indicated by the yellow dash line). Blue arrows indicate the linear trends observed in the inverse RSAM associated with gas pulses.

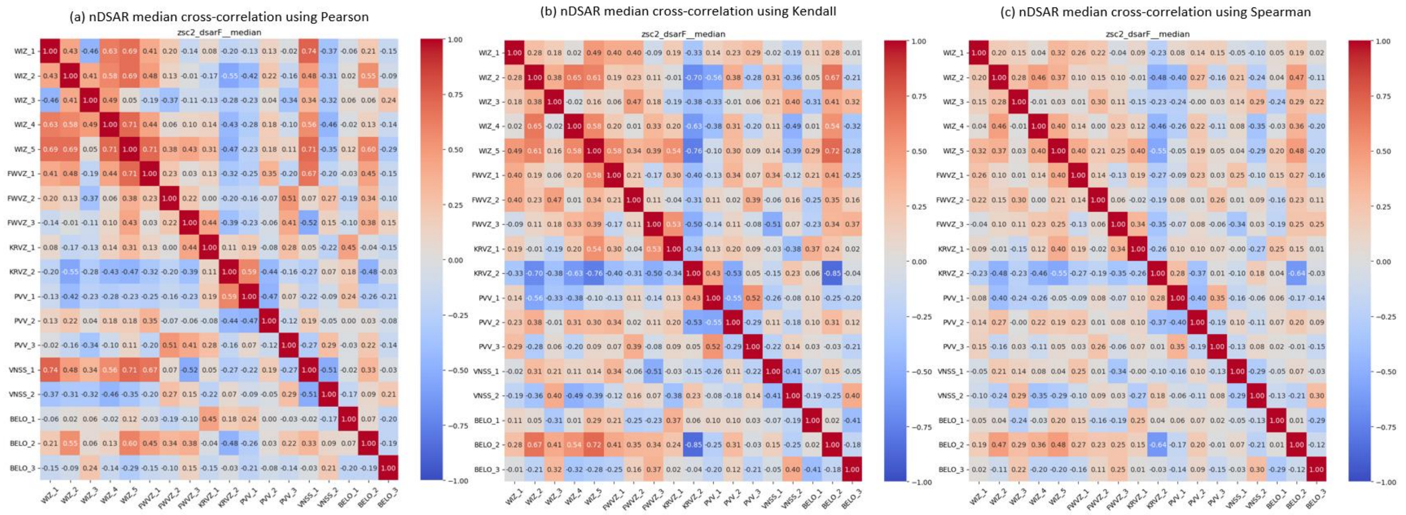

Figure S12. Matrix of correlation coefficients between eruption pairs for the four-week *nDSAR median* using (a) Pearson, (b) Spearman and (c) Kendall correlation methods.

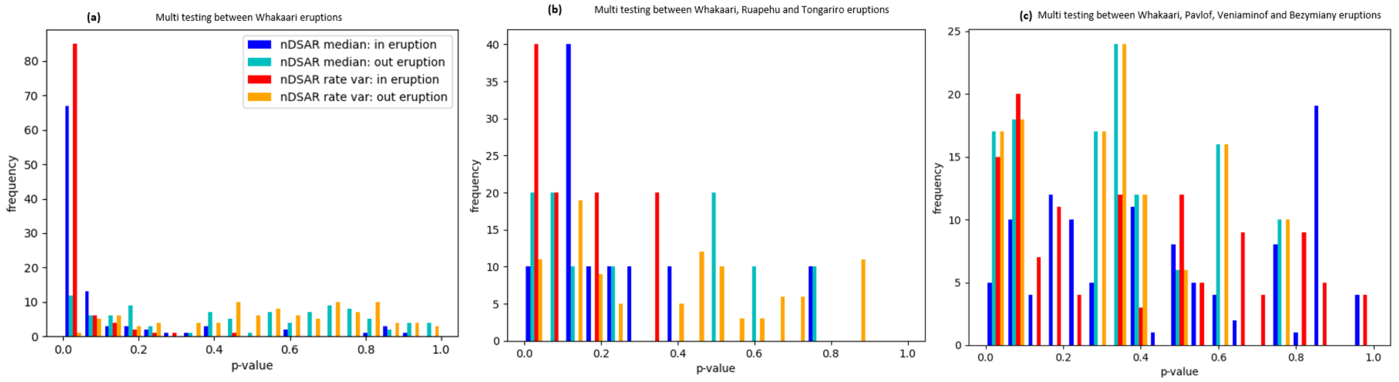

Figure S13. Statistical multi-testing and cross-validation of archetypes across different eruption pools. This is an equivalent version to Figure 3d-f in the main text, but here archetypes (see main text) are compared (correlated) non-overlapping signal (archetypes of 4 weeks long, are compared with 4 weeks signal sections that do not overlap between each other). (d) Five Whakaari eruptions. (e) Phreatic eruptions from Whakaari, Ruapehu and Tongariro. (f) phreatic Whakaari and magmatic Veniaminof, Pavlof and Bezymianny eruptions. Distribution of K-S test statistics from repeat testing of *nDSAR median* and *nDSAR rate variance* archetypes prior to eruptions (denoted “in eruption”; blue & red) and randomly selected from the non-eruptive record (denoted “out eruption”; cyan & yellow). Increasing differentiability is indicated by a distribution that clusters closer to zero.
